# Analyzing the postulated inhibitory effect of Manumycin A on farnesyltransferase

**DOI:** 10.3389/fchem.2022.967947

**Published:** 2022-12-06

**Authors:** Anna Hagemann, Philipp Klemens Altrogge, Miriam Caroline Alice Kehrenberg, Daniel Diehl, Dominik Jung, Lea Weber, Hagen Sjard Bachmann

**Affiliations:** Institute of Pharmacology and Toxicology, Centre for Biomedical Education and Research (ZBAF), School of Medicine, Faculty of Health, Witten/Herdecke University, Witten, Germany

**Keywords:** farnesyltransferase, Manumycin A, apoptosis, prenylation, *Caenorhabditis elegans*

## Abstract

Manumycin A is postulated to be a specific inhibitor against the farnesyltransferase (FTase) since this effect has been shown in 1993 for yeast FTase. Since then, plenty of studies investigated Manumycin A in human cells as well as in model organisms like *Caenorhabditis elegans.* Some studies pointed to additional targets and pathways involved in Manumycin A effects like apoptosis. Therefore, these studies created doubt whether the main mechanism of action of Manumycin A is FTase inhibition. For some of these alternative targets half maximal inhibitory concentrations (IC_50_) of Manumycin A are available, but not for human and *C. elegans* FTase. So, we aimed to 1) characterize missing *C. elegans* FTase kinetics, 2) elucidate the IC_50_ and K_i_ values of Manumycin A on purified human and *C. elegans* FTase 3) investigate Manumycin A dependent expression of FTase and apoptosis genes in *C. elegans*. *C. elegans* FTase has its temperature optimum at 40°C with K_M_ of 1.3 µM (farnesylpyrophosphate) and 1.7 µM (protein derivate). Whilst other targets are inhibitable by Manumycin A at the nanomolar level, we found that Manumycin A inhibits cell-free FTase in micromolar concentrations (K_i human_ 4.15 μM; K_i__*C. elegans*_ 3.16 μM). Furthermore, our gene expression results correlate with other studies indicating that thioredoxin reductase 1 is the main target of Manumycin A. According to our results, the ability of Manumycin A to inhibit the FTase at the micromolar level is rather neglectable for its cellular effects, so we postulate that the classification as a specific FTase inhibitor is no longer valid.

## Introduction

Historically, Manumycin A was developed as an antimicrobial agent isolated from *Streptomyces pavulus* Tu64 (Yang et al., 1997). In 1993, it was described as a highly potent, specific farnesyltransferase (FTase) inhibitor (FTI) in yeast (Hara et al., 1993). The inhibitory effect seems to be based on its analogy to the prenyl substrate farnesylpyrophosphate (Carey et al., 2015).

FTase belongs to the group of prenyltransferases (FTase and geranylgeranyltransferase I-III (GGTase I-III)) (Schaber et al., 1990; Boguski et al., 1992; Kuchay et al., 2019), performing an important post-translational modification in eukaryotes. Canonically, prenylation leads to membrane association and functionality of about 200 proteins in cell signalling pathways (Willumsen et al., 1984; Xu et al., 2015b). FTase catalyses the post-translational attachment of a farnesyl (15C) moiety to the cysteine of the so called CAAX-box at the C-terminus of proteins (Goossens et al., 2005; Lane and Beese, 2006; Cox et al., 2015; Wang and Casey, 2016). Well-known farnesylated proteins are the small GTPases of the Ras-superfamily including Rab, Ran, Ras, Rho and Arf, influencing cellular processes like cell differentiation and inflammation (Song et al., 2019). Members of the Ras-family are known to play a crucial role in tumorgenesis, explaining the extensive research on the drug class of FTIs and its member Manumycin A (Zhang and Casey, 1996; Gelb et al., 2006).

Most of the Manumycin A experiments have been carried out in cell cultures, as well as on *C. elegans*. Here, an age dependent reduction in motility was observed. Additionally, Manumycin A evoked toxic effects on the worm, which are supposed to be due to the farnesyltransferase inhibiting properties of Manumycin A (Bar et al., 2009; Bar and Gruenbaum, 2010).

Remarkably, a lot of publications describe effects of Manumycin A on additional targets in human cells. For example, Manumycin A inhibits Iκ-B kinase β or the thioredoxin-reductase 1 (Bernier et al., 2006; Tuladhar and Rein, 2018). Manumycin A also seems to have an influence on phospho-protein kinase B (pAKT), PI3K (phosphoinositol 3 -kinase) and specificity protein 1 (Sp1) (Cho et al., 2015; Zhang et al., 2016). Several studies showed that Manumycin A is effective against different cancer types (Li et al., 2014) e.g., prostate cancer. Here, Manumycin A induced apoptosis in LNCaP cells, which was based on the intrinsic activation of Caspase-9 and -3 (Cas-9, Cas-3). Additionally, an upregulation of B-cell lymphoma 2 (Bcl-2) and a downregulation of Bcl-2 associated X protein (Bax) could be observed. Besides these pro-apoptotic effects, Manumycin A seems to have antiatherosclerotic, antibacterial, anti-inflammatory, antimycotic, anti-neurogenerative and hepatoprotective properties (Sattler et al., 1998; Arenz et al., 2001; Sugita et al., 2007; Saha and Nandi, 2009). All these findings create doubt that Manumycin A is just an FTI. So, we analyzed the postulated role of Manumycin A as a farnesyltransferase inhibitor and determined its kinetic parameters. Additionally, we reviewed the different effects described and postulate a new main way of action for Manumycin A explaining all previous findings.

## Materials and methods

### Cloning and heterologous expression in *Escherichia coli*

The *Escherichia coli* strains DH5α (Thermo Fisher Scientific, Waltham, MA, USA), and Rosetta (DE3)pLysS (Novagen, Darmstadt, Germany) for cloning and expression studies were cultured under standard conditions following the instructions of the manufacturers. The cloning of the coding regions of *C. elegans* FTα (*ceFNTA*, NM_001392242.1) and FTβ (*ceFNTB*, NM_001392654.1) in pETDuet1 (Novagen) was performed by BioCat (Mannheim, Germany). pETDuet1 harbors two multiple cloning sites with one comprising a 6xHIS-tag. In both co-expression plasmids, the β-subunit is cloned in frame with an N-terminal His-tag. Cells were grown in LB-medium at 37°C containing 50 µg/ml ampicillin. Expression was induced by addition of 0.4 mM isopropyl-1-thio-β-D-galactopyranoside at an OD_600_ of ∼ 0.6 and 0.5 mM ZnSO_4_. After induction cells were grown for 4 h at 34°C (Zimmerman et al., 1998), harvested by centrifugation (8,000×g, 4°C, 20 min) and stored at -80°C.

### Purification of recombinant enzymes

Affinity chromatography with Ni-NTA was used to co-purify His-tagged FTβ together with FTα. *E. coli* cells were suspended (5 ml/1 g wet cell weight) in buffer A (50 mM Tris/HCl, pH 7.5, 300 mM KCl, 10 mM MgCl_2_, 10 µM ZnCl_2_, 20 mM imidazole, 5 mM DTT) and protease inhibitor cocktail (Roche, Basel, Switzerland) and lysed by sonification (3 × 5 min) in an ice ethanol bath. Cell debris and intact cells were removed by centrifugation (21100 x g, 45 min, 4°C). The lysate was applied to a Ni-NTA IMAC (immobilized metal ion affinity chromatography) column (Qiagen, Hilden, Germany) equilibrated with 3 × 5 ml of buffer A. The flow through was applied five times to the column by gravity flow. After washing (3 × 10 ml buffer A by gravity flow) the column was incubated on ice for 5 min with 200 µl buffer AE (buffer A, 250 mM imidazole) and eluted by gravity flow.

### Immuno-blot and -detection

Protein fractions were analyzed by SDS-PAGE and infra-red immuno blot (LiCor, Lincoln, NE, USA) with a coupled specific His-infra-red antibody (6x-His-tag antibody, 1:2000, DyLight 680, Thermo Fisher Scientific, Waltham, MA, USA). After semi-dry blotting (30 min, 25 V) the nitrocellulose membrane was blocked (RT, 60 min, shaking) in blocking solution (LiCor), incubated with His-infra-red antibody (1:2000, blocking solution, 0.1% Tween20, RT, 60 min, shaking), washed (3 × 5 min, PBS, 0.1% Tween20, and 1 × 5 min PBS), dried in the dark and visualized on an Odyssey imager (LiCor).

### Continuous fluorescence assay

Enzyme activity of FTase was analyzed using a continuous fluorescence assay (Goossens et al., 2005). The assay was performed with enzyme purified *via* IMAC in black flat 96-well plates (Thermo Scientific) with a total volume of 250 µL.

Different concentrations (see determination of K_m_-/IC_50_-values) of peptide substrate Dansyl-GCVLS were preincubated with 5 mM DTT in H_2_O (total volume 50 µL) for 30 min at room temperature. Preincubated Dansyl-GCVLS/DTT solution and 120 µL of enzyme solution (2 µg enzyme in H_2_O) were incubated in assay buffer (final buffer concentrations of 50 mM Tris-HCl pH 7.5, 10 µM ZnCl_2_ and 0.03% n-Dodecyl-ß-D maltoside) for 3 min at 30°C. The reaction was started by adding 30 µL of farnesylpyrophosphate (FPP)/H_2_O solution in varying FPP concentrations (see determination of K_m_-/IC_50_-values). The increase in fluorescence was measured every 30 s for 60 min (extinction: 340nm, emission: 505 nm). All measurements of human FTase were performed at 30°C. For the determination of the optimal reaction temperature of *C. elegans* FTase, the enzyme was measured at varying temperatures using a Tecan microplate reader (Tecan Infinite 2000 PRO mPlex, Männedorf, Tecan group Ltd., Switzerland) from 7–40°C. Since the readers maximal temperature is 40°C, we performed heat precipitation with Ni-NTA purified enzyme. Therefore, *ce*FTase was incubated at 45°C and 50°C, respectively for 20 min and afterwards centrifuged for 30 min at 16000xg. The soluble fractions were measured as described and analyzed with the resulting pellet fractions by an SDS-PAGE.

### Determination of apparant K_M_-values

For the determination of the apparant K_M_-value for Dansyl-GCVLS, the concentration of the substrate was varied (0.125–16 µM) while the concentration of FPP was kept constant at a saturating substrate concentration of 10 µM. Accordingly, for the K_M_ of FPP, concentration of FPP was varied (0.125–16 µM), while the concentration of Dansyl-GCVLS was constant at 8 µM.

### Determination of IC_50_-values

Furthermore, half-maximal inhibitory concentrations were determined for the inhibitor Manumycin A with the continuous fluorescence assay at the optimal temperatures of the responding enzyme (30°C for human and 40°C for *ce*FTase). Substrate concentration of Dansyl-GCVLS (8 µM) and FPP (2 µM) were constant. For *ce*FTase Manumycin A was tested at final concentrations of 0.1–1,000 µM. With respect to *h*FTase, Manumycin A was tested at final concentrations of 0.1–200 µM. Manumycin A was dissolved in DMSO; the final concentration of DMSO did not exceed 5% in the assay. The initial reaction rates from the series of enzyme assays were calculated at varying inhibitor concentrations and related to the reaction rate in absence of inhibitor. The residual enzyme activity was plotted against the inhibitor concentration. IC_50_ was analyzed with GraphPad Prism (Non-linear regression, Dose-Response Inhibition). Experiments were conducted three times each (*n* = 3).

### Cell culture

LNCaP and PC3 cells are human prostate adenocarcinoma cells. HEK 293 are human embryo kidney cells. All cell lines were kindly provided from the Institute of Pharmacogenetics, University Hospital Essen, Essen, Germany. Cell line genotyping determined the identity of the cell lines. LNCaP were cultured in RPMI, HEK 293 in DMEM and PC3 in 50% RPMI and 50% Ham´s F-12, all media contained 10% FBS and 100 units/ml Penicillin/Streptomycin (1%) in a humidified atmosphere at 37°C and 5% CO_2_. Cells were passaged every 3–4 days at a confluence of 90%.

### Cell viability assay (MTT)

For analysis of the cell viability, MTT (3-(4,5-dimethylthiazol-2-yl)-2,5-diphenyl tetrazolium bromide) assay (Merck Millipore, Burlington, Massachusetts, USA) was used. In a 96-well plate, 5,000 (LNCaP), 10,000 (PC3) or 15,000 (HEK293) cells per well were seeded and grown for 24 h. Then cells were incubated with Manumycin A in different concentrations (1–60 µM) for 48 h. After incubation, MTT assay was performed according to manufacturer`s protocol.

### Cultivation of *C. elegans*

Strain maintenance was performed under standard conditions on Nematode Growth Medium (NGM) agar as described by Brenner, 1974 (Brenner, 1974). *E. coli* OP50 streaked on the plates was used as feeding source. The wild-type strain N2 was used after three to four generations. The treatment with Manumycin A and DMSO respectively was performed as follows: feeding plates with 20 ml media were plated, inoculated and incubated with *E. coli* OP50 and afterwards coated with 3 μM, 9 μM, 25 μM, 50 μM or 100 μM Manumycin A as final concentration. The highest amount (0.55%) of DMSO was applied to plates, serving as negative control. As an additional control, the TrxR inhibitor D-9 was applied to the plates (300 nM, 100 nM, 30 nM and 10 nM). After drying, 1-3 worms were put on the plates, incubated for 6–7 days at 22°C, harvested and washed with phosphate buffered saline (PBS).

For the measurement of endogenous FTase from *C. elegans*, plates and worms were prepared as described above. To test for FTase inhibition by Manumycin A, 63 nM were applied to the plates; this is the concentration where a phenotype, but no toxicity was observed by Bar and Gruenbaum (Bar and Gruenbaum, 2010). Additionally, we cultivated non-treated as well as DMSO-treated (0.55%) *C. elegans*. Worms were harvested from ∼30 plates using PBS and washed in Lysis-Buffer (50 mM Tris/HCl, pH 7.5, 300 mM KCl, 10 mM MgCl_2_, 10 µM ZnCl_2_, 20 mM imidazole, 5 mM DTT and protease inhibitor cocktail (Roche, Basel, Switzerland)). After three freeze thaw cycles with liquid nitrogen and a 37°C water bath, the worms were mechanically lysed in a homogeniser and centrifuged (4°C, 16.000 x g, 45 min) (Kohl et al., 2018). The supernatant was used in the continuous fluorescent assay as described above. We determined the initial flow rate using GraphPad Prism Software (GraphPad Software 8.0, San Diego, CA, USA).

### RNA extraction and cDNA synthesis

*C. elegans* were washed from the plate harvested in 1 ml PBS and stored at -80°C overnight. After thawing, samples were homogenized using a rotor-stator homogenizer (TissueLyser II, Qiagen, Hilden) and total RNA preparation was performed using the QuickRNA-Kit (Zymo Research, Freiburg, Germany) according to the manufacturer’s instructions. RNA was quantified photometrically at 260 and 280 nm using a Tecan Infinite200 PRO plate reader. An OD_260nm/280nm_ of 1.9–2.1 was considered as protein free RNA (Supplementary Table S1). All RNA samples were stored at -80°C.

For cDNA synthesis, we used the PrimeScript RT Master Mix (Takara Bio, Kusatsu, Japan) according to the manufacturer’s instructions. For each cDNA synthesis, a quantity of 1 µg RNA was added to a volume of 4 µl of 5x PrimeScript RT Master Mix (Takara Bio) and stocked up to 20 µl with RNAse free ddH_2_O (Takara Bio). The thermocycler T100 (Bio-Rad Laboratories, Hercules, California) was set to a reverse transcription step of 15 min at 37°C, a 5 s inactivation step at 85°C and holding temperature at 4°C, according to the manufacturer’s protocol. The cDNA samples were immediately stored at -20°C.

### RT-qPCR protocol and assay validation

Adequate reference gene stability of *cdc42* was validated in adherence to the minimum information for publication of quantitative real-time PCR experiments (MIQE) guidelines by means of the Genorm algorithm (Bustin et al., 2009; Vandesompele et al., 2009). All PCR primers contain an exon-exon junction and have an amplicon length of 70–133 nucleotides (Supplemental Table S2). Oligonucleotides were obtained from Eurofins MWG Operon LLC (Eurofins Scientific SE, Luxemburg) and primer efficiency (E_p_) was determined by means of a 6x log_10_ serial dilution of a pooled standard cDNA solution of untreated *C. elegans*. In brief, reference and target genes were amplified and a standard curve was created by means of linear regression analysis in PRISM 8 software (GraphPad Software, San Diego, California). Coefficient of determination (r^2^) was calculated from the respective linear regression and E_p_ was derived from the slope: E_p_ = 10^−1/slope^ -1. Acceptable E_p_ and r^2^ thresholds within the linear dynamic range (LDR) were predefined at 90–110% and r^2^ > 0.97, respectively. The assay specificity for each gene of interest was evaluated by melt curve analysis, with a singular peak signifying primer specificity (Supplementary Figures S1, S2). Bias from contaminating genomic DNA or primer dimers was assessed with a no-template control (NTC) for each sample and a no-reverse-transcriptase control (NRT) containing RNA instead of cDNA.

For qPCR amplification, a CFX96 Touch Real-Time PCR cycler (Bio-Rad) was used with 96-well Hard-Shell PCR plates (Bio-Rad). 20 µl mastermix, consisting of 2x iTaq Universal SYBR^®^ Green Supermix (10 μl, Bio-Rad), the respective cDNA solution (1 µl), the respective primer pair (10 pmol/0.3 µl per primer) and nuclease-free H_2_O (8.4 µl, Fresenius Kabi, Bad Homburg, Germany) was administered per well. The plates were covered with Nunc sealing tape (Nalge Nunc International, Rochester, New York). Amplification of three biological replicates was carried out in technical triplicates for each gene of interest and every experimental condition. A C1000 Touch Thermal Cycler in conjunction with the CFX96 Real-Time System (Bio-Rad) was used. The amplification protocol (40 cycles, initial denaturation at 95°C/3 min, denaturation at 95°C/10 s, annealing at the respective T_a_/30 s, extension at 70°C/10 s) was followed by a consecutive melt curve analysis (65°C–95°C in 0.5°C increment/5 s each).

### *In silico* analysis and statistics

Blast analysis was performed using NCBI (Altschul et al., 1990; Altschul et al., 1997) as well as ClustalO (Sievers et al., 2011; Li et al., 2015). Statistical analysis was performed using GraphPad Prism version 7.00 for Windows (GraphPad Software, La Jolla, USA). For the determination of the optimal reaction temperature, we plotted the highest initial reaction rates against the temperature and presented as mean. For the determination of the K_M_, the initial reaction rates were plotted against the substrate concentration and fitted to the Michaelis-Menten equation using non-linear curve fitting of GraphPad Prism Software (GraphPad Software 8.0, San Diego, CA, USA). We calculated K_M_-values and its 95% confidence interval.

Based on the experimentally determined IC_50_ values, we calculated the inhibition constant (K_i_). The relationship is described by Cheng-Prusoff (Cheng and Prusoff, 1973). FTase acts *via* ordered sequential mechanism with FPP binding first (Ochocki et al., 2014). The following equation is applicable for Manumycin A competing with the first substrate in a bisubstrate enzyme mechanism:

$${IC}_{50}=K_{i}\left(1+\frac{\left[FPP\right]}{K_{m}\left(FPP\right)}\right)\left(1+\frac{\left[Dansyl-GCVLS\right]}{K_{m}\left(Dansyl-GCVLS\right)}\right)$$

The K_M_, K_I_ and IC_50_ values are presented as the mean with 95% confidence interval. Analysis of RT-qPCR data was performed by the common base method (Ganger et al., 2017). In brief, Cq values were corrected for amplification efficiency following the formula CqE = log10 (E_p_)*Cq and ΔCqE values were obtained by subtracting target gene values (CqE_GOI_) from the reference gene values (CqE_REF_). Next, ΔΔCqE was calculated by means of ANOVA and Tukey’s multiple comparisons test. Mean differences and 95% CI were transformed back (10^−ΔΔCqE^) to obtain the relative fold change. Mean differences resulting in p-values <0.05 were considered significant.

## Results

### Protein alignment of human and *C. elegans* FTase shows high conservation

To elucidate the comparability of *C. elegans* FTase with human FTase, we first evaluated the percentage identity of human and *C. elegans* FTase subunits. Therefore, we performed sequence alignments with ClustalO. The α-subunit has a query coverage of 79% with 40% identity and the β-subunit of 86% with 45.6% identity. This illustrates the high conservation of FTase throughout eukaryotes (Supplementary Figures S3, S4).

### *ce*FTase has a high temperature optimum and comparable K_M_s to *h*FTase

**FIGURE 1:**
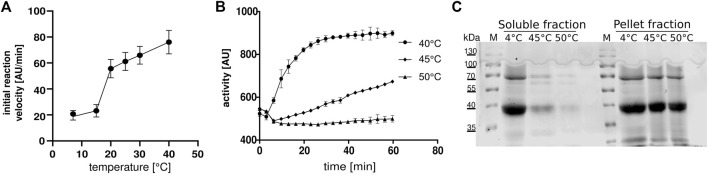
Temperature optimum of *ce*FTase **(A,B)** Continuous fluorescent *ce*FTase assays **(A)** Initial flow depended on temperature. **(B)** Activity of samples after heat precipitation, measured at 45°C and 50°C, the supernatant was measured at 40°C. **(C)** SDS-PAGE after heat precipitation. The 4°C fraction is the extract after Ni-NTA compared to the soluble fraction and the pellet fraction after heat precipitation. M: marker.

**FIGURE 2:**
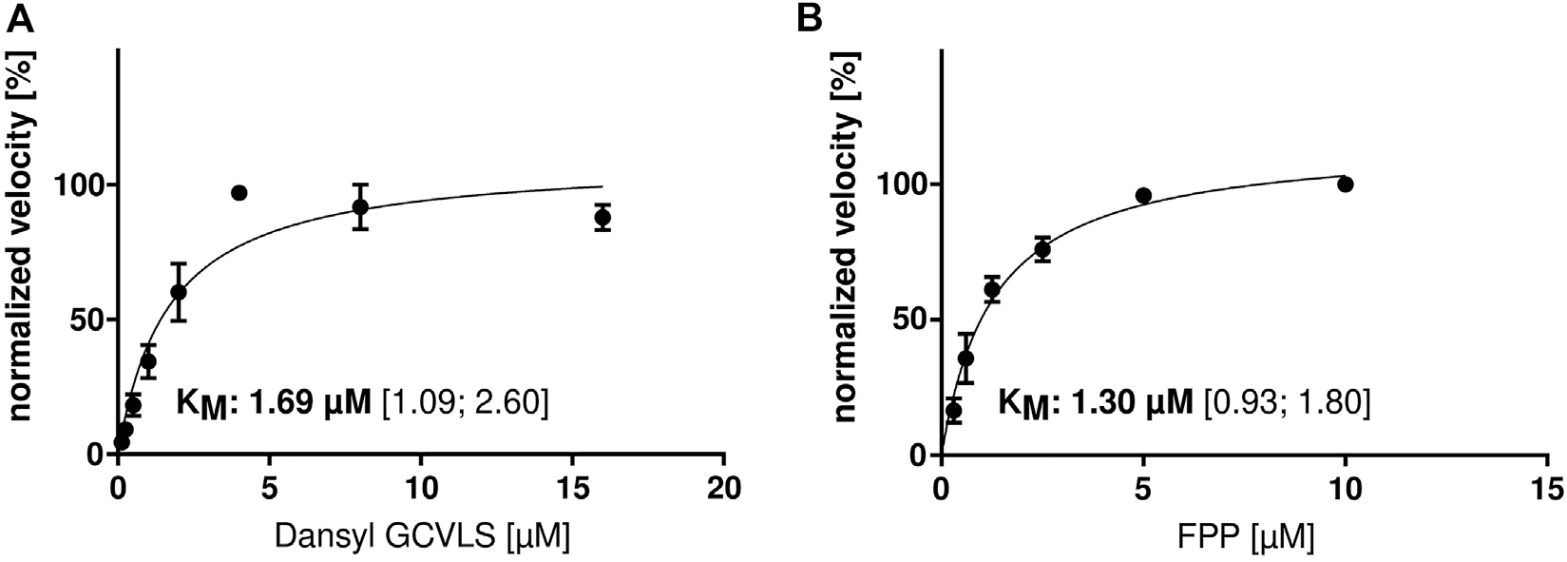
K_M_ determination of *ce*FTase. Determination of the K_M_ for **(A)** Dansyl-GCVLS (with fixed [FPP]) and **(B)** FPP (with fixed [Dansyl-GCVLS]). The change in fluorescence was measured and the mean ± SEM of the normalized velocity plotted against the substrate concentration. The K_M_ and the 95% confidence interval (CI) are given.

Since there are no published functional parameters for the FTase from *C. elegans*, we determined the optimal assay temperature for the enzyme assay. *h*FTase has an optimal temperature of 30°C (Goossens et al., 2005), but due to the optimal growing temperature of *C. elegans* at 20°C, we performed the assay at temperatures of 15 and 20°C. Interestingly, the activity was higher at 20°C so we performed the experiments with a wider temperature range (7, 15, 20, 25, 30 and 40°C). To our surprise, the highest activity could be measured at 40°C (Figures 1A,B). Heating up the assay to 45 and 50°C led to enzyme degradation (Figure 1C) and hardly any activity was measured. Having the optimal reaction temperature, we determined the K_M_s for FPP (1.3 μM) and Dansyl-GCVLS (1.7 μM) (Figure 2).

### Manumycin A does not totally inhibit LNCaP, HEK293 and PC3 viability

**FIGURE 3:**
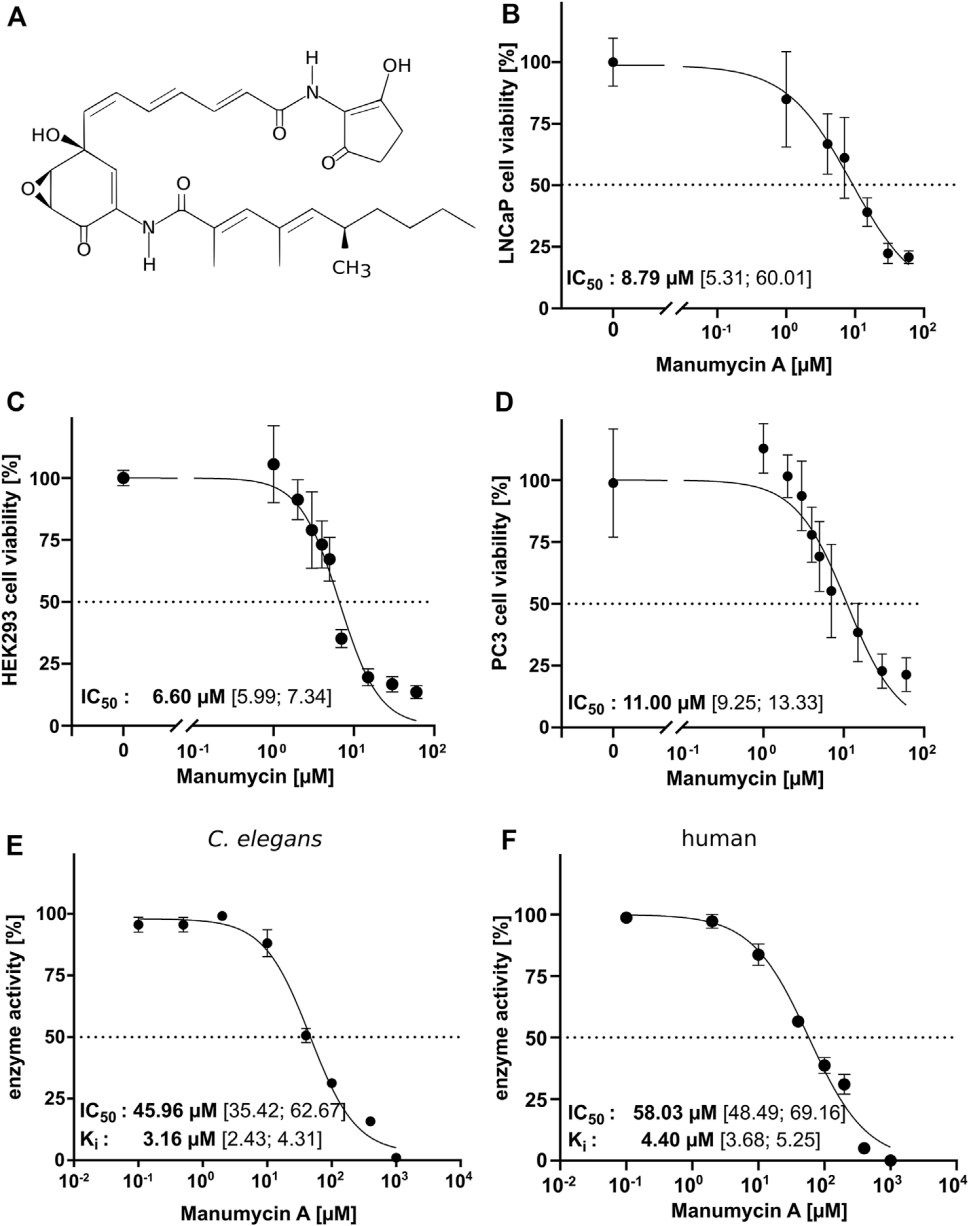
IC_50_ for Manumycin A in a cell-based and a cell-free assay **(A)** Chemical structure of Manumycin A (Cho et al., 2015), **(B–D)** Determination of the IC_50_ of Manumycin A in treated LNCaP, HEK293 and PC3 cells. IC_50_ and K_i_ was determined with constant concentrations of FPP and Danysl-GCVLS and increasing amounts of Manumycin A for **(E)** *ce*FTase and **(F)** human FTase. The 95% confidence interval (CI) is given in square brackets.

To analyze the inhibitory effect of Manumycin A (Figure 3A) in cell culture, cell viability of different cell lines (LNCaP, HEK293 and PC3) was tested after incubation with increasing amounts of Manumycin A for 48 h. The determined IC_50_s are 8.79 µM (LNCaP), 6.60 µM (HEK293) and 11.00 µM (PC3) (Figures 3B–D). Our results are in line with the findings of Li et al. showing a reduction of cell viability at a concentration of 30 µM Manumycin A down to 20% (Li et al., 2014). Doubling the concentration of Manumycin A does not lead to a further decrease of viability.

### No inhibition of *C. elegans* endogenous FTase after treatment with Manumycin A

To evaluate whether Manumycin A treatment leads to an inhibition of the endogenous *ce*FTase, we exposed the worms to 63 nM Manumycin A, the concentration, where Bar and Gruenbaum first identified a phenotype after treatment that was not toxic (Bar and Gruenbaum, 2010). The initial flow rates of *ce*FTase in the whole cell lysate from untreated worms, DMSO-treated worms (0.55%) and Manumycin A treated worms (63 nM) were akin to each other (untreated: 7.154 (95% CI 6.47-7.83), DMSO: 7.86 (95% CI 7.21-8.51) and Manumycin A: 8.11 (95% CI 7.40-8.82)) (Supplementary Figure S5).

### High IC_50_ values for Manumycin A in FTase cell-free assay

To investigate whether the described effects of Manumycin A in cell culture assays could depend on the inhibition of FTase or are related to other mechanisms in the cells, we determined the IC_50_ for human and *C. elegans* IMAC purified FTase using increasing Manumycin A concentrations at the optimal assay temperature for human (30°C) and *ce*FTase (40°C). For *h*FTase, we determined an IC_50_ of 58.03 μM and a K_i_ of 4.40 μM (CI 3.68–5.25 µM)—for *ce*FTase the IC_50_ value is 45.96 μM and the Ki 3.16 μM (CI 2.43–4.31 µM) (Figures 3E,F). The high IC_50_ values indicate that a quite high concentration of Manumycin A is needed to inhibit the FTase. Around 8.8 µM Manumycin A is needed to have a 50% reduction in cell viability. This is five times less compared to the cell-free assay. Interestingly, when measuring the IC_50_ of *ce*FTase at a much lower temperature (25°C), we got an even higher value with 117.3 µM (CI 69.3–191.3 µM) (Supplementary Figure S6).

### Manumycin A but not the TrxR inhibitor D-9 up-regulates *fnta* and *fntb* gene transcription in a dose dependent manner in *C. elegans*

**FIGURE 4:**
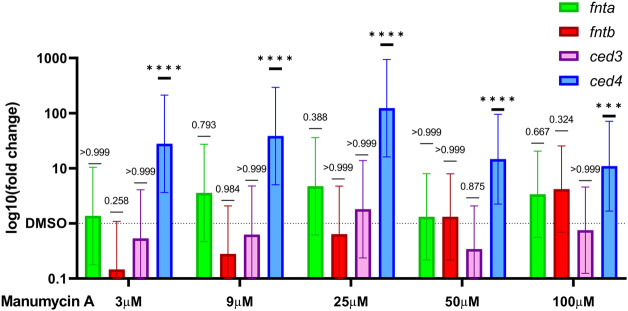
Change in the relative expression of FTase and apoptosis related genes. Relative expression of *fnta* and *fntb* and *ced-3* and *ced-4* after treatment with 3 μM or 100 μM of Manumycin A. Graphs represent the log10 relative fold changes (10^−ΔΔCq^) normalized to a respective DMSO control group. Cq values were corrected for amplification efficiency following the formula CqE = log10 (E_p_)*Cq. ∆∆CqE was calculated by means of ANOVA and Tukey’s multiple comparisons test. Mean differences and 95% CI were transformed back (10^-∆∆CqE^) to obtain the relative fold change. *p < 0.05, **p < 0.01, ***p < 0.001.

To elucidate whether expression of the farnesyltransferase genes *fnta* and *fntb* is influenced by the addition of Manumycin A, RT-qPCR was performed. The worms were exposed to either 3, 9,25, 50 or 100 μM of Manumycin A or DMSO. The data show that relative gene expression of farnesyltransferase genes *fnta* and *fntb* was upregulated in response to the varying doses of Manumycin A (Figure 4). Moreover, *fntb* transcription was up-regulated in a dose-dependent manner. After incubation, the expression of *fnta* increased 1.3-fold (3 µM) to 4.7-fold (25 µM) in response to Manumycin A. However, the mean *fnta* expression in DMSO treated and Manumycin A treated groups were not significantly different. *fntb* expression increased dose-dependently from initial downregulation by 0.15-fold up to a 4.2-fold upregulation. The mean differences between the worms treated with 100 µM Manumycin A and the DMSO treated worms were statistically not significant either (Figure 4). These results indicate a dose dependent influence on *C. elegans* farnesyltransferase expression but only at very high Manumycin A concentrations, supporting the findings from the cell-free assay. In response to treatment with various concentrations of the TrxR inhibitor D-9, neither *fnta* nor *fntb* transcription exhibited considerable upregulation. On the contrary, *fnta* was significantly down-regulated by D-9 in concentrations of 10, 30 and 300 nM (Supplementary Figure S7).

### Manumycin A induces *C. elegans* apoptosis pathways *via* cell-death protein 4

In addition to the farnesyltransferase genes *fnta* and *fntb,* we investigated two genes that are involved in *C. elegans* apoptosis, namely cell death protein 3 and 4 (CED-3 and CED-4). CED-3 is an analogous to the human apoptotic protease activating factor-1 (APAF-1) and CED-4 to Caspase-9. In response to Manumycin A, the relative gene expression of *ced4* was significantly up-regulated in a dose-dependent manner from 3 to 25 µM (27-fold–123-fold), followed by a 14.7-fold and 10.9-fold up regulation for 50 μM and 100 μM Manumycin A, respectively (Figure 4). On the other hand, *ced3*, a Caspase analogue, was not or inversely regulated by Manumycin A. Similarly, the effect of D-9 led to a significant increase of *ced4* transcription, ranging from 12-fold to 16-fold, accompanied by a non-significant down-regulation of *ced3* (Supplementary Figure S6)*.* From the data obtained, one could assume that this effect relies on a CED-3 independent apoptotic event in response to both, Manumycin A and D-9 (Bloss et al., 2003; Chen et al., 2008).

## Discussion

This is the first time that kinetic data for *ce*FTase are determined. Surprisingly, *ce*FTase shows its maximal activity at 40°C, even though the worm´s cell cycle, cell division and reproduction are negatively affected by temperatures above 25°C (Begasse et al., 2015). Although *C. elegans* has thermosensory neurons, all tests with it have only been performed up to 25°C. In this study the authors proposed that the thermosensory system helps *C. elegans* to withstand higher temperatures (Lee and Kenyon, 2009). However, a study by Gómez-Orte et al. could show, that *C. elegans* shows not only a changed transcriptome by temperature alterations but also by the kind of applied diet (Gómez-Orte et al., 2017). Worms fed on *E. coli* show no regulation of FTase independent of the temperature. When fed on *Bacillus subtilis*, a transcriptional upshift of *fnta* could be observed at 15°C (Gómez-Orte et al., 2017). The high temperature optimum for *ce*FTase is not a uniqueness. This could also be observed for malat dehydrogenases 1 and 2 of *C. elegans* having their maximal activity at 40°C and 35°C, respectively (Thomas et al., 2022).

The K_M_ values for FTase that can be found in literature differ quite widely depending on the organism and the assay used. The value for Dansyl-GCVLS from *Rattus norvegicus* (0.7 μM) is comparable with 1.7 μΜ for *C. elegans* (Reigard et al., 2005)*.* The K_M_-values for FPP found in the literature range from 0.2 nM in *Plasmodium falciparum* (Eastman et al., 2007) to 46 μΜ for *Rattus norvegicus* (Micali et al., 2001).

So far, no kinetic data on the inhibitory property of Manumycin A as an FTI existed. With our data, we can demonstrate that Manumycin A is capable of inhibiting human or *C. elegans* FTase in a cell-free assay, but not in pharmacologically relevant concentrations. The IC_50_ and K_i_ values are relatively high (IC_50_ 58.03 μM/K_i_ 4.40 µM for human and IC_50_ 45.96 μM/K_i_ 3.16 µM for *C. elegans* FTase). Lonafarnib and tipifarnib, the most prominent FTIs, show 24000 times lower IC_50_ values of 1.9 nM and 0.86 nM, respectively (Curtin et al., 2003; Puntambekar et al., 2007). These FTIs are not competitive inhibitors against FPP like Manumycin A, but against the CAAX substrate. Other known FPP analogues like α-hydroxyfarnesylpyrophosphate show 850-fold lesser values (K_i_ of 5.2 nM) in bovine brain (Gibbs et al., 1993).

Since many studies prove Manumycin A to induce apoptosis, we investigated the effect *in vitro* using LNCaP, HEK293 and PC3 cells. Li et al. showed that the cell viability of LNCaP cells is reduced to 20% after treatment with 30 μM Manumycin A (Li et al., 2014). Our findings are in line with that and with that of other studies showing IC_50_ values in different cell lines between 4.3 and 50 µM Manumycin A (She et al., 2005; Cho et al., 2015; Giudice et al., 2016; Kim et al., 2016; Sojka et al., 2021). Like Li et al., we were not able to induce a complete inhibition of cell growth, even when using concentrations of 100 μM Manumycin A. Importantly, the concentrations to reduce cell viability to 50% were far below those required to inhibit FTase in our cell-free assay, indicating that Manumycin A results in apoptosis independent of FTase inhibition. Furthermore, we did not observe an inhibition of endogenous FTase in the worms’ whole cell lysate after Manumycin A treatment.

On the RNA level, the cell-free determined IC_50_ concentration (50 µM Manumycin A) leads to no relevant regulation of *fnta* and *fntb.* Only after doubling the Manumycin A concentration, we see a slide upshift of the RNA expression of both genes. However, we can only see a dose-dependent increase of the *fntb* expression, but not for *fnta,* leading to the question, whether this is a direct effect of the Manumycin A treatment or just a side effect. It could be shown, that treatment with 10 µM of the highly potent FTI-276 reduces the FTase activity but upregulates the expression von FNTA and FNTB in human rheumatoid synovial fibroblasts (Abeles et al., 2007). Manumycin A leads to the activation of apoptotic genes, seen by a 14.7-fold upregulation of *ced4*. To our knowledge, *ced4* is not directly connected to treatment with Manumycin A. Therefore, we expanded our literature research on effects of Manumycin A independent of FTase inhibition.

Various studies have highlighted single aspects of Manumycin A influencing apoptotic pathways, like TrxR-1, NF-κB, pAKT, PI3K, the Bcl-2 family as well as the proteasome, to name just a few (Frassanito et al., 2002; Bernier et al., 2006; Zhang et al., 2016; Tuladhar and Rein, 2018; Mofers et al., 2020). In contrast, most studies using Manumycin A as an FTI refer to Hara et al. (Hara et al., 1993) who described the reagent to be a highly potent and specific FTI in yeast, but some studies doubt the role of Manumycin A as an FTI (Storck et al., 2019).

**FIGURE 5:**
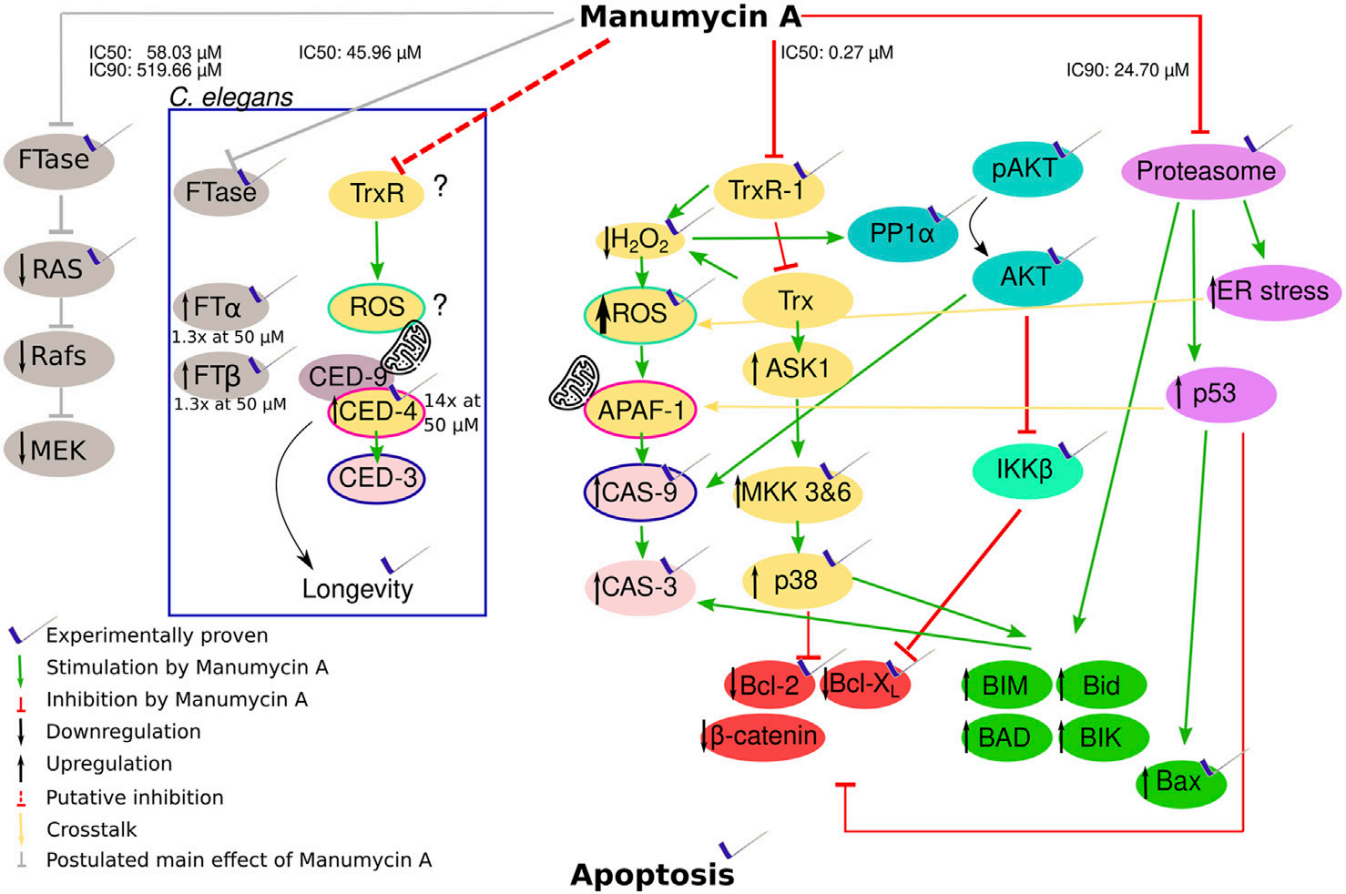
Hypothesis of how Manumycin A acts with the proven or probable influence on apoptotic metabolic pathways. Yellow and pale red part: Manumycin A inhibits the TrxR-1 enzyme, a major player in the mammalian redox system. The inhibition decreases the intracellular H_2_O_2_ and increases the intracellular reactive oxygen species (ROS). This leads to an activation of caspase-9 and caspase-3 triggering apoptosis. The increasing ROS levels lead to the oxidation of Trx that cannot be reduced by TrxR1 due to its inhibition. The oxidized Trx detaches from ASK1, promoting the proapoptotic proteins and downregulating the antiapoptotic proteins *via* several intermediate steps. Turquoise part: The lower H_2_O_2_ levels cause a reduced inhibition of PP1α, resulting in a dephosphorylation of Akt that no longer can exert its anti-apoptotic activity. Caspase 9 is significantly less inhibited. Light green part: The β-subunit of the IκB kinase complex is inhibited, whereby the NF-κB inhibitor α is no longer phosphorylated and the transcription factors of the propapoptotic proteins Bcl-2 and Bcl-X_L_ are less functional. Pink part: The proteasome is inhibited by Manumycin A, resulting in less degradation of proapoptotic proteins. An increased ER stress additionally promotes an increase in ROS. Green part: Proapoptotic proteins influenced by the different pathways. Red part: Antiapoptotic proteins influenced by the different pathways. Grey part: Manumycin A inhibits FTase and therefore p21Ras, leading to the inhibition of the following pathway. Part in the box: TrxR and FTase inhibition in *C. elegans* with the genes corresponding to humane genes.

To our knowledge, this is the first paper that brings prominent effects of Manumycin A in a context, explaining most of the effects and showing that its role as FTI is neglectable for apoptosis in cancer cells (Figure 5). It was shown that Manumycin A inhibits thioredoxin reductase 1 (TrxR1) with an IC_50_ of 272 nM, resulting both in accumulation of reactive oxygen species (ROS) and in activation of the xenobiotic apoptotic pathway (Xu et al., 2015a; Tuladhar and Rein, 2018). The latter comprises apoptosis regulating kinase 1 (ASK1), p38 mitogen activated protein kinase (MAPK)-pathway as well as c-Jun NH2-terminal Kinase (c-JNK) (Cheng et al., 2014) and it has been shown that this xenobiotic apoptotic pathway is upregulated after treatment with 54 μM Manumycin A (Saitoh et al., 1998; She et al., 2006; Fujino et al., 2007). The accumulation of ROS on the other hand leads to inhibition of the NF-κB pathway and thus to reduced expression of anti-apoptotic proteins like Bcl-XL or Baf (Rothwarf et al., 1998). Other groups showed that BclXL is downregulated by Manumycin A (Li et al., 2014) resulting in less anti-apoptotic proteins. This downregulation of BclXL and upregulation of Bax was also observed by Kim et al. after the addition of 2.5–10 µΜ Manumycin A (Kim et al., 2016). In line with that, Bernier et al. postulated, that 10 μM Manumycin A inhibits selectively IKKβ, an upstream regulator of NF-κB (Bernier et al., 2006). Irrespective of NF-κB inhibition, accumulation of ROS in response to Manumycin A was also observed by Sears et al. (Sears et al., 2008) leading to the activation of Cas-9 and Cas-3, triggering apoptosis as well. In line with that, the effect of Manumycin A on ROS elevation and H_2_O_2_ loss was also shown by Carey et al. after an administration of 4 μM Manumycin A (Carey et al., 2015). An upregulation of Cas-3 after Manumycin A treatment could also be observed in other studies (Frassanito et al., 2002).

Furthermore, another important effect of Manumycin A is the induction of chaperone expression as well as the induction of endoplasmic reticulum stress (ER stress). It was shown that Manumycin A increases the amount of polyubiquitinated proteins and inhibits the proteasome (Mofers et al., 2020). All this leads to the expression of the proapoptotic protein NOXA that is normally degraded by the proteasome. Due to the higher amount of NOXA, Caspase-9 is activated, leading subsequently to apoptosis (Qin et al., 2005; Ploner et al., 2008).

Interestingly these data from human cell lines are in line with our experimental results regarding the regulation of the *C. elegans* cell death proteins *ced-3* and *ced-4* (Figure 5, inner box)*. ced-3* is the corresponding gene to the human *cas-9* and *ced-4* to *apaf*, respectively (Nakajima and Kuranaga, 2017) (Figure 5)*.* In both organisms, APAF/CED-4, induce CAS-9/CED-3. In contrast to the human system, it was shown, that elevated mitochondrial ROS levels in *C. elegans* result in longevity by activating the BH3-only protein and therefore CED-9 and CED-4 (Yee et al., 2014). Accordingly, Manumycin A treatment prolongs *C. elegans* motility (Bar and Gruenbaum, 2010). The expression of *ced-4* was 15-fold upregulated after Manumycin A treatment. Yee at al. also proposed that CED-3 is not as important as CED-4 for this reaction reflected by our findings that there is no expressional regulation of *ced-3* after Manumycin A treatment.

Summarizing all these data, we hypothesize that Manumycin A is neither a highly potent nor a specific FTI. We rather propose that it has multi-plane effects on the highly cross-linked network of apoptotic pathways. From our point of view, the inhibition of TrxR1 is the main key player inducing apoptosis *via* different pathways that influence each other. On the one hand, there is an increase in ROS and a decrease in H_2_O_2_, highly having impact. On the other hand, ASK1 cannot be inhibited which induces apoptosis. All the mentioned effects lead to the activation of caspases and subsequently to apoptosis. The exact mechanisms how all these rack-wheels interact need to be elucidated in further studies, taking our findings on the role of Manumycin A as new starting point.

## Data Availability

The original contributions presented in the study are included in the article/Supplementary Material, further inquiries can be directed to the corresponding author.
