# Supplementary material for: Analyzing the postulated inhibitory effect of Manumycin A on farnesyltransferase
Source: Front Chem. 2022 Dec 6;10:967947. doi: 10.3389/fchem.2022.967947 (PMC9763582; doi:10.3389/fchem.2022.967947)
Supplement: Supplementary file 1 [file DataSheet1.docx]

**Supplements**

**Supplementary Table 1 Summary of the *C. elegans* RNA quality for qPCR**

RNA quantity and OD_260/280_ ratio of *C. elegans* treated with Manumycin A dissolved in DMSO in concentrations of 3 to 100 μM (A), D-9 in concentrations of 10-300 nM (B) or the corresponding amount of DMSO (0.225 and 0.55 %). RNA quantity and purity were measured spectrophotometrically for each biological replicate.

Rep. = biological replicate, OD = optical density


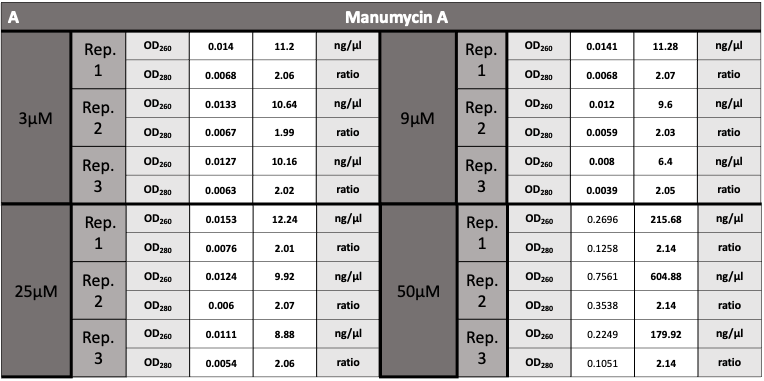


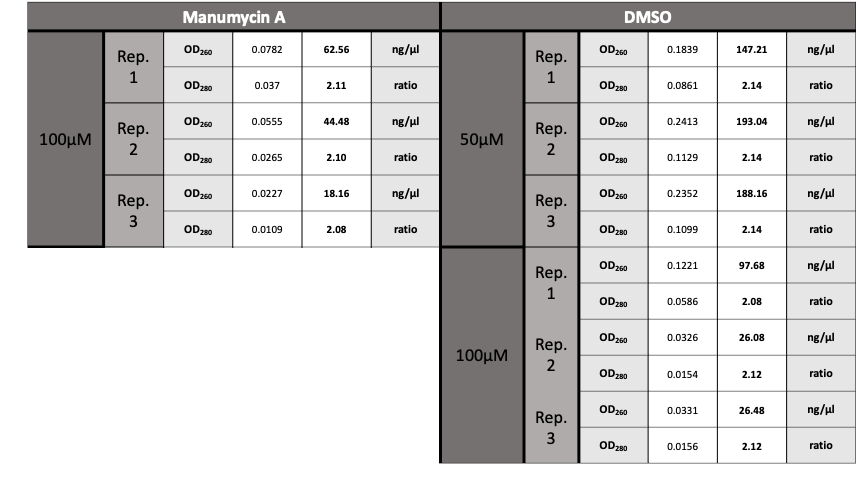


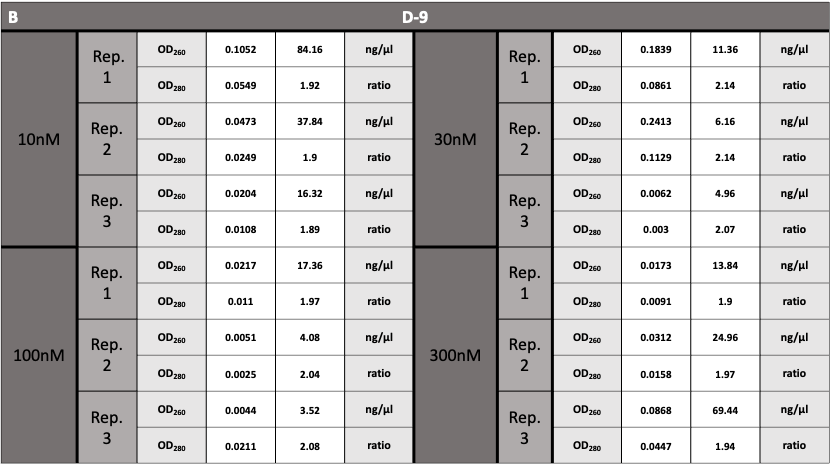


**Supplementary Table 2 Summary of the *C. elegans* primer sets for R-qPCR**

Target names, function, primer sequence in 5´-3´ direction with melting temperature (T_m_) and GC-content, amplicon length in bases and amplification efficiency is given

| **Gene** | **Sequence number**  **(Wormbase)** | **Function** | **5′→3′ Forward primer (T_m_/%GC)** | **5′→3′ Reverse primer**  **(T_m_/%GC)** | **Amplicon length [b]** | **Amplification**  **efficiency** |
| --- | --- | --- | --- | --- | --- | --- |
| ***fntb-1*** | F23B12.6 | Farnesyltransferase β-subunit | CTCGCTTCTTAAAGACGTCCC  (58.74°C/52.38%) | CGGGTGGAAAACATAATTTAGGC  (58.32°C/43.48%) | 70 | 2.04 |
| ***fnta-1*** | R02D3.5 | Farnesyltransferase  α-subunit | AATTGCACGGAAGTAGGCGA  (59.12°C/55.00) | AATTGCACGGAAGTAGGCGA  (60.04°C/50.00%) | 96 | 1.98 |
| ***cdc-42*** | R07G3.1 | Cell division cycle related | CTCTCGACCCACCACAACAG (60.32°C/60.00%) | GAGAAGAGTGGAAGTCGGGG  (59.47°C/60.00%) | 80 | 2.02 |
| ***ced-3*** | C48D1.2 | Cell Death related | AGATGACATCCCGCCTGCTC  (62.04°C/ 60.00%) | TGGGCGAAAGAGAACTGGGG  (62.12°C/60.00%) | 133 | 1.92 |
| ***ced-4*** | C35D10.9 | Cell Death related | TTCTTTCCTCTTCCCCTTG (60.16°C/36.00%) | ACGCCAACAGAGGAAAGAAATCG  (61.66°C/47.83%) | 97 | 1.78 |


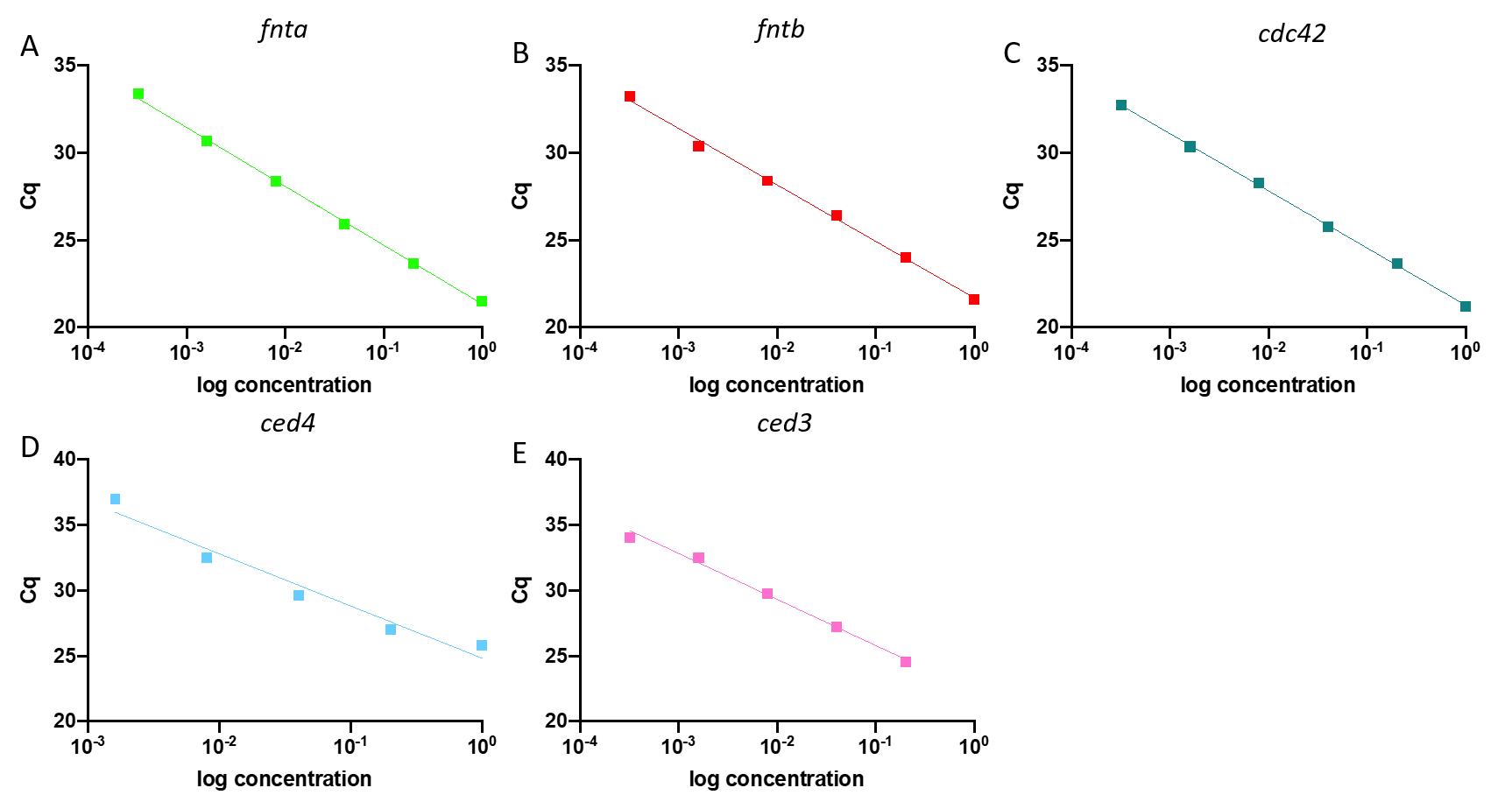


**Figure S1 Standard curves of the respective primer pairs for *fntb* (A), *fnta* (B), *cdc42* (C), *ced3* (D) and *ced4* (E).**

Primer efficiency (Ep) was determined by means of a 6x log10 serial dilution of a pooled standard cDNA solution of untreated *C. elegans.* The reference and target genes were amplified and a standard curve was created by means of linear regression analysis in PRISM 8 software (GraphPad Software, San Diego, California). The coefficient of determination (r2) was calculated from the respective linear regression and primer efficiency (Ep) was derived from the slope: Ep= 10-1/slope -1. Acceptable Ep and r2 thresholds within the linear dynamic range (LDR) were predefined at 90-110% and r2 >0.97, respectively.


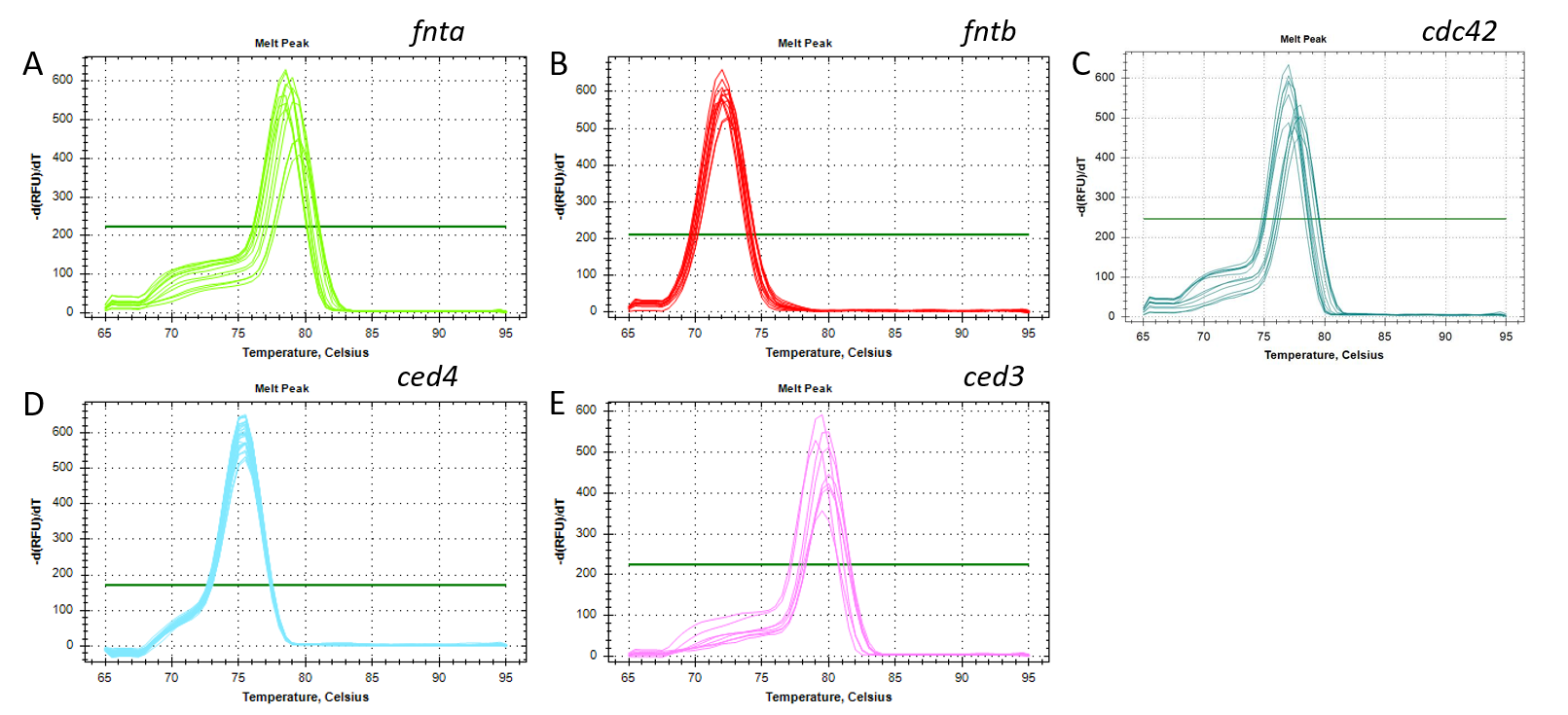


**Figure S2 Melt curve analysis of amplicons to measure product specificity of targets *fntb* (A), *fnta* (B), *cdc42* (C), *ced3* (D) and *ced4* (E).** The melt curve analysis was performed immediately after the last RT-qPCR cycle. Each replicate well was heated incrementally in steps of 0.5°C with a consecutive fluorescence measurement. A single peak of the melt curve indicates homogenous melting temperature and thus product specificity. The green line indicates the fluorescence threshold.

ceFTα ------------------------------------------------------MSDSDI 6

hFTα MAATEGVGEAAQGGEPGQPAQPPPQPHPPPPQQQHKEEMAAEAGEAVASPMDDGFVSLDS 60

: . *

ceFTα PSSTLYKDNVDWKDITPIYPSKEEEVAVKIAVTEDFTDAFAYFRAILIKNEKSDRVMALL 66

hFTα PSYVLYRDRAEWADIDPVPQNDGPNPVVQIIYSDKFRDVYDYFRAVLQRDERSERAFKLT 120

** .**:*..:* ** *: .. : .*:* ::.* *.: ****:* ::*:*:*.: *

ceFTα EDCIRLNPANYTVWQYRRVCLTELGWDLKKEMRYLSDIIQESPKNYQVWHHRRFIVETIG 126

hFTα RDAIELNAANYTVWHFRRVLLKSLQKDLHEEMNYITAIIEEQPKNYQVWHHRRVLVEWLR 180

.*.*.** ******::*** *..* **::**.*:: **:*.***********.:** :

ceFTα ESAVNDELHFCSEVIRDENKNYHAWQHRQWVVRTFKVPLEKELTFALHMLLLDNRNNSAY 186

hFTα DP--SQELEFIADILNQDAKNYHAWQHRQWVIQEFKLW-DNELQYVDQLLKEDVRNNSVW 237

: .:**.* ::::.:: ************:: **: ::** :. ::* * ****.:

ceFTα NYRYFLMTLYDKTEDASQLDIEINLAKKFIENIPNNESAWNYLAGLLITNGVTSNSDVVS 246

hFTα NQRYFVISNTTGYNDRAVLEREVQYTLEMIKLVPHNESAWNYLKGILQDRGLSKYPNLLN 297

* ***::: :* : *: *:: : ::*: :*:******** *:* .*::. :::.

ceFTα FVEDLYETTPEEKRSPFLLAFIADMMLENIENQKSAEESAG-RAKKLYKDL-QSIDPVRV 304

hFTα QLLDLQ----PSHSSPYLIAFLVDIYEDMLENQCDNKEDILNKALELCEILAKEKDTIRK 353

: ** .: **:*:**:.*: : :*** . :*. :* :* : * :. * :*

ceFTα NYYRHQSLLAQTMLIK---AQTKVTAK 328

hFTα EYWRYIGRSLQSKHSTENDSPTNVQQ- 379

:*:*: . *: . : *:*

**Figure S3 Aminoacid alignment of *Caenorhabditis elegans*** **and *Homo sapiens* FTase α-subunits**

Alignment with *Caenorhabditis elegans* and Homo sapiens Farnesyltransferase α-subunit (ceFTα and hFTα). The reference sequences used are NP_001380029.1 for ceFTα and NP_002018.1 for hFTα. The alignment was performed by the webtool ClustalO. An * (asterisk) indicates positions with a single, fully conserved residue. A : (colon) indicates conservation between groups of strongly similar properties. A . (period) indicates conservation between groups of weakly similar properties (Goujon, McWilliam et al. 2010, Sievers, Wilm et al. 2011, McWilliam, Li et al. 2013)

ceFTβ MTSSI----------------PF---RSFNEKYECNDDDNFTYSSTEQKRIETMLFENYN 41

hFTβ MASPSSFTYYCPPSSSPVWSEPLYSLRPEHARERLQDDSVETVTSIEQAKVEEKIQEVFS 60

*:* *: * : : . :**. * :* ** ::* : * :.

ceFTβ SLVLEPFKTTSDEDLAELTIFRQKHASYLLRYLKNCPSSYATLDASRSWMCYWGVNALKI 101

hFTβ SYKF-------NHLVPRLVLQREKHFHYLKRGLRQLTDAYECLDASRPWLCYWILHSLEL 113

* : :. : .*.: *:** ** * *:: .:* ***** *:*** :::*::

ceFTβ LDAEIPNDVIENIIVFLKSCEHPEGGYGGGPGQLAHLAPTYAAVMCLVSLQKEEALRSIN 161

hFTβ LDEPIPQIVATDVCQFLELCQSPEGGFGGGPGQYPHLAPTYAAVNALCIIGTEEAYDIIN 173

** **: * :: **: *: ****:****** ********* .* : .*** **

ceFTβ RVTLFNFLKKCKHESGGFYMHEGGEIDMRSAYCALATCEIVGLPMDEISNGVAEWIISCQ 221

hFTβ REKLLQYLYSLKQPDGSFLMHVGGEVDVRSAYCAASVASLTNIITPDLFEGTAEWIARCQ 233

* .*:::* . *: .*.* ** ***:*:****** :...:..: :: :*.**** **

ceFTβ SFEGGFGGEPYTEAHGGYTFCAVASLVLLNRFRLADMEGLLRWATRRQMRFEGGFQGRTN 281

hFTβ NWEGGIGGVPGMEAHGGYTFCGLAALVILKRERSLNLKSLLQWVTSRQMRFEGGFQGRCN 293

.:***:** * *********.:*:**:*:* * :::.**:*.* ************ *

ceFTβ KLVDGCYSFWQGAIFPLLDGEMEREGR---SLEKGLFEARMLEEYILVGCQSVHGGFKDK 338

hFTβ KLVDGCYSFWQAGLLPLLHRALHAQGDPALSMSHWMFHQQALQEYILMCCQCPAGGLLDK 353

***********..::***. :. :* *:.: :*. : *:****: **. **: **

ceFTβ PDKPVDLYHTCYVLSGLSVAQKYSLAR--DGKILGGDVNMLAEINPVFNVTIASEQFAKE 396

hFTβ PGKSRDFYHTCYCLSGLSIAQHFGSGAMLHDVVLGVPENALQPTHPVYNIGPDKVIQATT 413

*.* *:***** *****:**::. . .. :** * * :**:*: . *.

ceFTβ FFTSH------------------- 401

hFTβ YFLQKPVPGFEELKDETSAEPATD 437

:* .:

**Figure S4 Aminoacid alignment of *Caenorhabditis elegans*** **and *Homo sapiens* FTase β-subunits**

Alignment with *Caenorhabditis elegans* and Homo sapiens Farnesyltransferase α-subunit (ceFTβ and hFTβ). The reference sequences used NP_001379157.1 for ceFTβ and NP_002019.1. The alignment was performed by the webtool ClustalO. An * (asterisk) indicates positions with a single, fully conserved residue. A : (colon) indicates conservation between groups of strongly similar properties. A . (period) indicates conservation between groups of weakly similar properties (Goujon, McWilliam et al. 2010, Sievers, Wilm et al. 2011, McWilliam, Li et al. 2013)


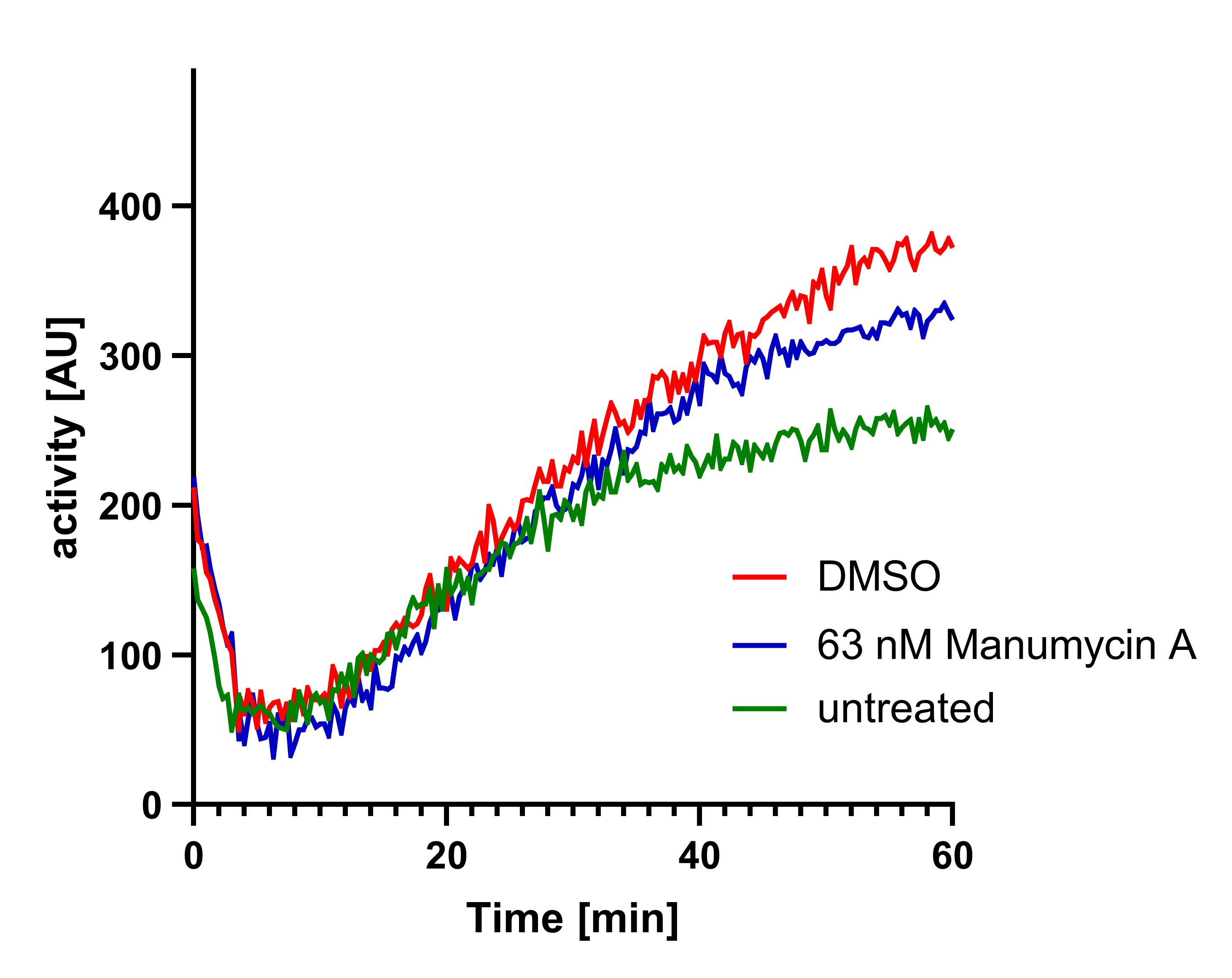


**Figure S5 Inhibition of endogenous *C. elegans* FTase after Manumycin A treatment**

Continuous fluorescent assays with endogenous *ce*FTase measured at 40°C.

**Figure S6 IC_50_ for Manumycin A in a cell-free assay at 25°C**

IC_50_ was determined with constant concentrations of FPP and Danysl-GCVLS and increasing amounts of Manumycin A for *ce*FTase at suboptimal 25°C. The 95 % confidence interval (CI) is given in big brackets.


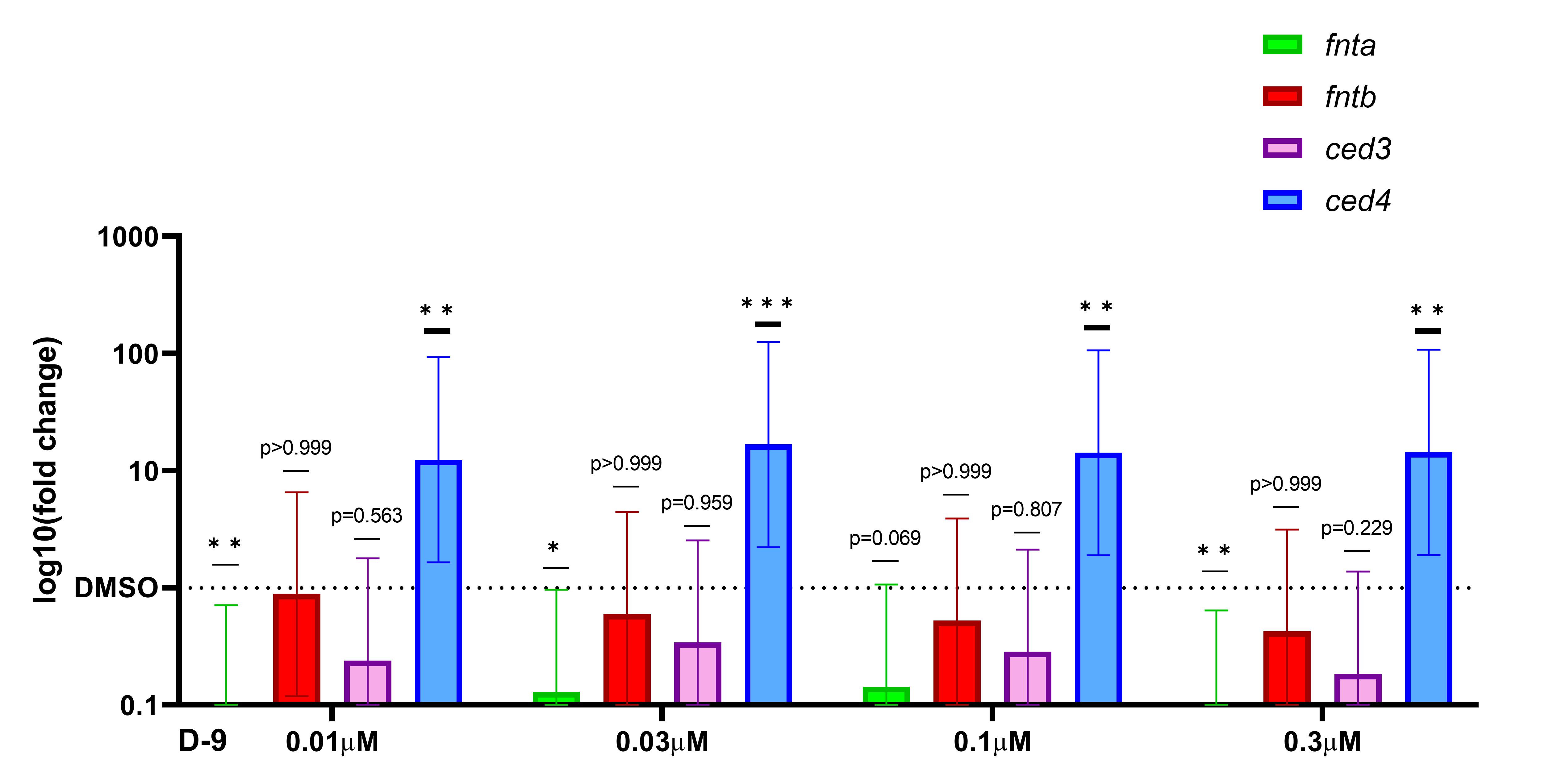


**Figure S7** **Change in the relative expression of FTase and apoptosis related genes after D-9 treatment**

Relative expression of *fnta* and *fntb* and *ced-3* and *ced-4* after treatment with either 0.01 -0.03 μM of D-9. Graphs represent the log10 relative fold changes (10^-𝛥𝛥Cq^) normalized to a respective DMSO control group. Cq values were corrected for amplification efficiency following the formula CqE=log10(E_p_)*Cq. ∆∆CqE was calculated by means of ANOVA and Tukey’s multiple comparisons test. Mean differences and 95% CI were transformed back (10^-∆∆CqE^) to obtain the relative fold change. *p<0.05, **p<0.01, ***p<0.001

Goujon, M., H. McWilliam, W. Li, F. Valentin, S. Squizzato, J. Paern and R. Lopez (2010). "A new bioinformatics analysis tools framework at EMBL–EBI." Nucleic Acids Research **38**(suppl_2): W695-W699.

McWilliam, H., W. Li, M. Uludag, S. Squizzato, Y. M. Park, N. Buso, A. P. Cowley and R. Lopez (2013). "Analysis Tool Web Services from the EMBL-EBI." Nucleic Acids Research **41**(W1): W597-W600.

Sievers, F., A. Wilm, D. Dineen, T. J. Gibson, K. Karplus, W. Li, R. Lopez, H. McWilliam, M. Remmert, J. Söding, J. D. Thompson and D. G. Higgins (2011). "Fast, scalable generation of high-quality protein multiple sequence alignments using Clustal Omega." Molecular Systems Biology **7**(1): 539.
